# A modular verification and validation framework for hybrid-capture next-generation sequencing HLA typing in clinical histocompatibility laboratories

**DOI:** 10.3389/fgene.2026.1959391

**Published:** 2026-10-12

**Authors:** Alice Scarpa, Diego Ferrarese, Samantha Guidotti, Veronica Scaglia, Angela Rossi

**Affiliations:** Transplant Biology, Molecular Diagnostics and HSC Manipulation Unit, Azienda USL di Piacenza, Piacenza, Italy

**Keywords:** healthcare, histocompatibility testing, HLA, next-generation sequence (NGS), quality assurance, risk managemenent

## Abstract

Hybrid-capture next-generation sequencing HLA workflows are increasingly used in clinical histocompatibility laboratories for high-resolution HLA typing. Compared with amplicon-based approaches, they may provide broader coverage of HLA genes, improved ambiguity resolution and the possibility of including additional targets; however, their greater analytical, bioinformatic and interpretative complexity requires a clear definition of intended use, reportable scope and required validation level. A practical and modular framework was developed for the verification and validation of hybrid-capture next-generation sequencing HLA workflows, applicable to IVDR-regulated methods used according to the manufacturer’s instructions for use, locally adapted IVDR workflows, research-use-only workflows converted to clinical use, laboratory-developed or laboratory-adapted workflows and outputs not fully covered by manufacturer claims. Core modules required for clinical HLA typing are distinguished from additional modules activated by specific claims or local adaptations, including analytical performance, bioinformatic validation, risk management and reporting governance. It provides an operational pathway to document fitness for intended use and to distinguish clinically reportable results from technically available outputs not validated for diagnostic use. A dataset generated under routine conditions using a commercial full-length HLA capture-based workflow is used as a practical implementation example. A modular structure integrating analytical performance, bioinformatic control, risk-based thinking, reporting governance and continuous monitoring is provided by this framework in clinical immunogenetics and histocompatibility laboratories.

## 1 Introduction

Next-generation sequencing has substantially changed HLA typing in clinical histocompatibility and immunogenetics laboratories. High-resolution HLA characterization is essential for donor-recipient selection in hematopoietic stem cell transplantation and is increasingly relevant in selected solid organ transplantation settings, as well as for selected HLA-disease association applications and increasingly complex immunogenetic contexts. Compared with different approaches, NGS methodologies allow a broader analysis of polymorphic regions, improved phasing, and reduced ambiguity, while introducing new analytical and interpretative complexity (Hosomichi et al., 2015; Weimer et al., 2016).

Among available NGS approaches, hybrid-capture workflows enable enrichment of extended genomic regions and may include classical HLA loci, non-classical HLA loci and, in some cases, additional genomic targets. Compared with amplicon-based approaches, the literature highlights several potential advantages of hybrid-capture workflows. Coverage is generally more uniform across the entire gene, including introns and regulatory regions, with less dependence on the efficiency of individual primer pairs. The risk of allele dropout caused by mismatches at primer annealing sites is reduced, which is particularly relevant for highly polymorphic loci such as HLA. Hybrid-capture approaches also offer a greater ability to detect structural variants, insertions/deletions and novel variants not anticipated by primer design, together with improved resolution of phase ambiguities and greater flexibility in panel updates without the need to redesign numerous amplicons. However, these advantages are associated with greater analytical and bioinformatic complexity and more stringent validation requirements. This broader analytical capability makes a clear definition of the intended use and a structured validation process essential (Hosomichi et al., 2015; Wittig et al., 2015).

Although most analytical performance characteristics are shared across NGS-based HLA typing approaches, hybrid-capture workflows introduce enrichment-specific sources of variability that should be explicitly considered during verification and validation. These include capture efficiency, on-target rate, coverage uniformity, locus- or allele-specific enrichment bias, potential effects of sequence variation within probe-binding regions, and sensitivity of enrichment performance to DNA input, DNA quality, and hybridization conditions. The proposed framework therefore applies general NGS verification and validation principles while specifically integrating risks associated with hybridization-based target enrichment.

Hybrid-capture HLA NGS workflows can be implemented in the clinical laboratory through different pathways. Some assays are implemented as commercial IVDR-regulated devices according to the manufacturer’s instructions for use; others are RUO products, locally adapted workflows, or combinations of commercial reagents, sequencing platforms, analysis software and laboratory-defined interpretative rules. Therefore, a validation model cannot rely solely on the commercial status of the product, but should be driven by the intended use, clinical impact, any local modification, sample type, reportable targets, the role and influence of the analysis software on final results, and applicable accreditation or regulatory requirements (European Parliament and Council, 2017).

In accredited settings, method validation must be embedded within a quality management system. ISO 15189:2022 provides the general framework for quality and competence in medical laboratories, with particular emphasis on examination procedures, risk management, validity of results, process control, personnel competence and continuous monitoring. Professional histocompatibility standards, including EFI Standards and other applicable national or international requirements, add specific requirements for HLA typing, allelic ambiguities management, participation in external quality assessment programmes and interpretative competence. ISO 5649:2024 also provides useful concepts for laboratory-developed, laboratory-adapted, RUO or off-IFU workflows, particularly when the laboratory introduces different sample sources, clinical applications, software components or interpretative claims not fully covered by the manufacturer’s instructions (International Organization for Standardization, 2022; International Organization for Standardization, 2024).

A practical, modular and transferable framework for the validation of hybrid-capture HLA NGS workflows is proposed in this work. The framework is not linked to a single kit, manufacturer, or accreditation scheme, but is intended to provide a structure that can be adapted to different regulatory and professional contexts. The objective is to support laboratories in defining a validation pathway that integrates analytical performance, data quality, validation of bioinformatic software, risk management, reporting limits, and post-implementation monitoring.

## 2 Framework development and standards mapping

A modular validation framework was developed for hybrid-capture next-generation sequencing HLA typing workflows in clinical histocompatibility laboratories. The framework was designed to be applicable across different implementation scenarios, including IVDR-regulated workflows used according to the manufacturer’s instructions for use, locally adapted IVDR workflows, research-use-only workflows converted to clinical use, laboratory-developed workflows, off-IFU applications and outputs not fully covered by manufacturer claims.

Framework development was based on three preliminary elements: definition of the intended use, classification of the workflow according to regulatory status and local adaptation, and identification of the reportable scope. The intended-use definition included clinical indication, reportable loci, required resolution, accepted sample source and type, analytical platform, interpretation software, report format, method limitations and personnel competencies required for test performance and result review.

The reportable scope was defined as the set of analytical outputs used for clinical reporting, established in accordance with the intended use and in consultation with the relevant clinical users. It should specify, as applicable, the HLA loci and genomic regions included, reporting resolution, ambiguity policy, accepted specimen types, software/database version, relevant limitations, and conditions requiring confirmatory testing or restricted reporting.

The framework was primarily mapped to the EFI Standards for Histocompatibility and Immunogenetics Testing, version 9.0, for H&I-specific requirements and then aligned with ISO 15189:2022 quality and competence requirements. Applicable concepts from ISO 5649:2024 were considered for laboratory-developed, laboratory-adapted, RUO or off-IFU workflows. Additional guidance relevant to HLA NGS and diagnostic NGS bioinformatics was considered for software validation, database control, pipeline verification and post-implementation monitoring (5,6,10).

For the purposes of this framework, workflows were grouped into five operational categories: commercial IVDR-regulated workflows used according to the manufacturer’s IFU (C1); commercial IVDR-regulated workflows with local adaptations (C2); RUO workflows implemented for clinical reporting (C3); laboratory-developed or laboratory-adapted workflows (C4); and exploratory or research outputs not intended for clinical reporting (C5) (see Figure 1; Table 1). This classification was used to determine the expected extent of local verification or validation.

**FIGURE 1:**
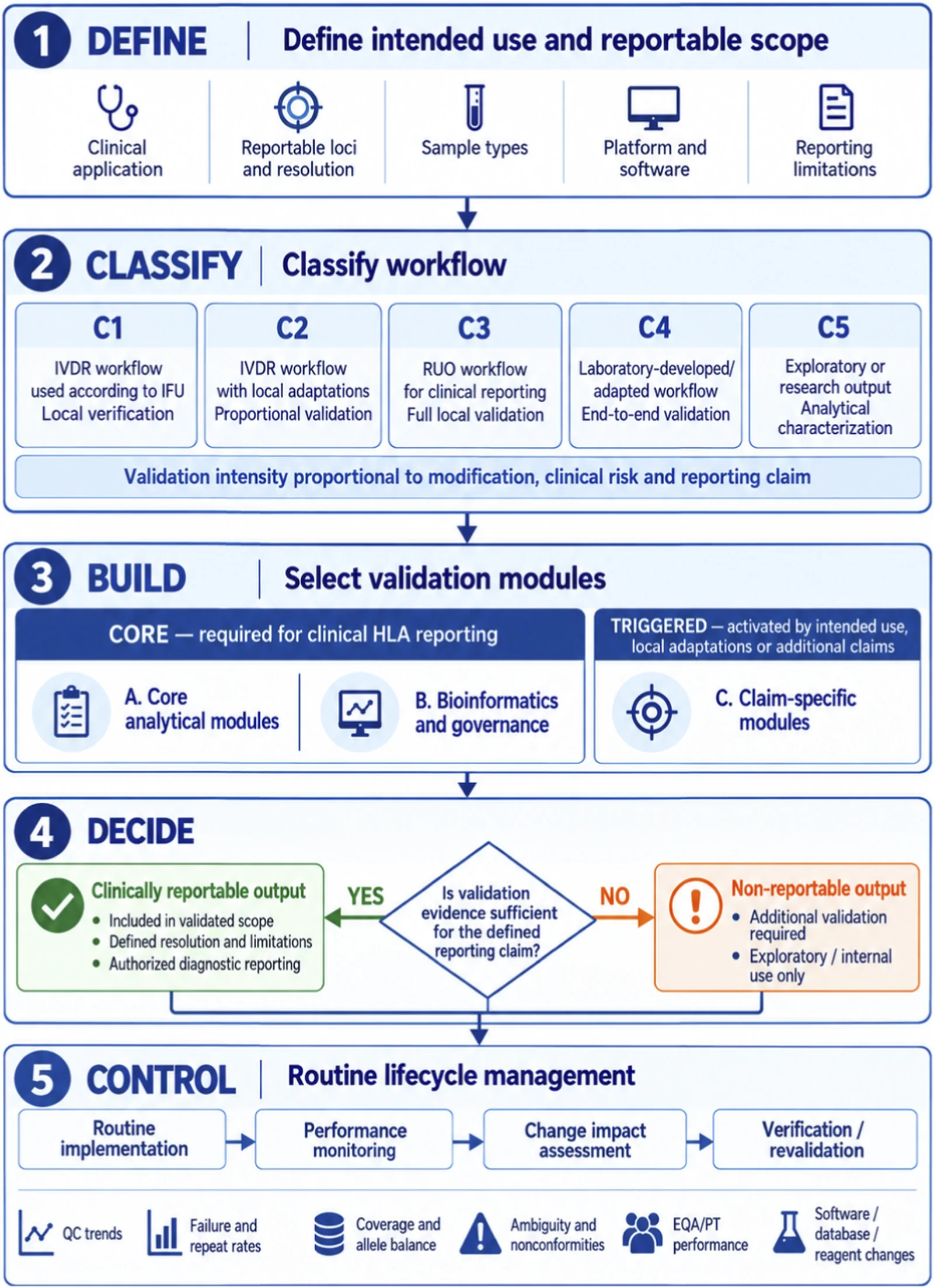
Modular validation framework for hybrid-capture NGS HLA typing.

**TABLE 1:** Workflow categories and recommended verification or validation approach.

Workflow category	Typical situation	Recommended validation approach	Relevant framework
C1: Commercial IVDR-regulated workflow used according to IFU	Commercial kit used as labeled for the intended sample type, instruments, software and claims	Local verification of manufacturer claims, implementation, QC, staff competence evaluation and reporting framework	H&I standards; ISO 15189; local regulations
C2: Commercial IVDR-regulated workflow with local adaptation	Alternative sample type, lower DNA input, different platform/software, additional loci or modified interpretation	Partial or full validation of modified components, proportional to risk assessment analysis	H&I standards; ISO 15189 plus ISO 5649 concepts for adapted/off-IFU components; local regulations
C3: RUO workflows implemented for clinical reporting	RUO capture assay implemented in a diagnostic workflow	Full local validation, risk assessment, software validation and clinical reporting governance	H&I standards; ISO 15189; ISO 5649/LDT lifecycle concepts; local regulations
C4: Laboratory-developed or laboratory-adapted workflow incorporating commercial components	Commercial reagents combined with local bioinformatics, local reporting rules or non-manufacturer claims	Validation of the complete end-to-end workflow	H&I standards and ISO 15189; software validation principles; ISO 5649 concepts where applicable
C5: Research or exploratory output only	Non-classical loci, CNV signals or HLA loss signals not clinically reported	Analytical characterization only; no diagnostic reporting unless specifically validated	Internal governance; research policy; future validation plan

For the purposes of this framework, verification refers to the confirmation that an established examination procedure achieves the expected performance under local laboratory conditions and within its defined intended use. Validation refers to the demonstration that a new, modified, laboratory-developed, or off-claim examination procedure is fit for its intended use through the generation of appropriate objective evidence. The extent of verification or validation should be proportional to the degree of modification, analytical uncertainty, clinical risk, and reporting claim.

### 2.1 Verification and validation framework design

The validation design should be organized into core and additional modules. Core modules were defined as those required to support routine clinical reporting of HLA genotypes and include analytical performance, pre-examination requirements, bioinformatic and database control, reporting governance, risk management, and external quality assessment/proficiency testing.

Additional modules were defined as risk-based or claim-specific modules activated when the laboratory extends the workflow beyond the manufacturer’s claims or standard intended use. These included RUO kits used for clinical reporting, off-IFU sample types, validated DNA input range and low-input robustness, degraded or archived DNA, saliva or buccal swab samples, post-transplant mixed-profile samples, non-classical HLA loci, software-derived copy number signals or HLA-loss indicators, ABO or non-HLA targets included in the same workflow, customized bioinformatics and additional reporting claims not covered by the manufacturer.

For each module, the purpose of the assessment, the expected documentation, the main performance or quality indicators, the relevant formulas used for metric calculation when applicable, and the required interpretation or reporting limitations were defined. The overall principle was that every reported element should be supported by validation or verification evidence consistent with the intended use. Conversely, technically available outputs not validated for clinical use should be excluded from diagnostic reports or clearly delimited as exploratory, internal or research data.

## 3 Verification and validation framework

### 3.1 Modular verification and validation workflow

A stepwise modular workflow was developed for the verification, validation and implementation of hybrid-capture NGS HLA typing in clinical histocompatibility laboratories. The framework begins with definition of the intended use and reportable scope, including the clinical application, reportable loci and resolution, accepted sample types, analytical platform, software components and reporting limitations.

The workflow is then classified according to its regulatory status and degree of local adaptation. This classification determines the expected extent of local verification or validation, ranging from verification of an IVDR-regulated workflow used according to the manufacturer’s instructions to full end-to-end validation of RUO, laboratory-developed or laboratory-adapted workflows.

Validation activities are organized into core and claim-specific modules. Core modules support routine clinical HLA reporting and include analytical performance, bioinformatic control, database management, risk management, reporting governance and EQA/PT. Additional modules are activated when the intended use includes local adaptations, non-standard sample conditions, extended targets or outputs not fully covered by the manufacturer’s claims.

The accumulated validation evidence is subsequently reviewed to determine whether each output can be included within the clinically reportable scope. Outputs not supported by sufficient evidence require further validation or remain restricted to exploratory or internal use. Following implementation, continued fitness for intended use is maintained through performance monitoring, change-impact assessment and proportional verification or revalidation as part of lifecycle control (Figure 1).

### 3.2 Standards-based modular mapping across workflow categories

The framework was mapped across the five operational workflow categories by integrating H&I-specific requirements with ISO 15189:2022 quality and competence requirements. ISO 5649:2024 concepts were applied where laboratory-developed, laboratory-adapted, RUO-to-clinical or off-IFU components were included. Through this mapping, verification and validation modules are defined as mandatory, triggered by local adaptation or additional claims, or exploratory unless specifically validated for clinical reporting (Table 2).

**TABLE 2:** Standards-based modular mapping of validation requirements across workflow categories.

Validation area	H&I-specific requirement	ISO 15189/ISO 5649 alignment	Applicability across workflow categories
Intended use and reportable scope	H&I application, loci, resolution, nomenclature and reporting context must be defined before clinical reporting.	ISO 15189: fitness for intended use, examination procedure scope and user needs. ISO 5649: intended-use definition for laboratory-developed, laboratory-adapted, RUO-to-clinical or off-IFU workflows.	All categories: required. C5: define non-diagnostic/exploratory scope.
Sample/template quality and pre-examination control	Template purity, quantity, quality and suitability must support interpretable NGS HLA typing.	ISO 15189: sample acceptance, traceability and pre-examination risk control. ISO 5649: verification or validation of modified sample types, matrices or input conditions when locally introduced.	C1: verify local implementation; C2: validate modified sample/input conditions; C3-4: Full validation; C5: Fit-for-purpose characterization.
Contamination and sample mix-up control	DNA contamination prevention, sample tracking, barcoding/pooling controls and traceability are core H&I/NGS requirements.	ISO 15189: risk management, nonconformity management, process control and records.	All clinical categories: core. Expanded challenge models may be added when mixed profiles or batching risks are clinically relevant.
Concordance and allele assignment	Criteria for allele assignment and agreement with reference or previously characterized HLA results must be documented.	ISO 15189: validation/verification evidence and validity of results. ISO 5649: performance specifications for laboratory-developed or laboratory-adapted components.	C1: local verification; C2: validate affected components; C3: full local validation; C4: end-to-end validation; C5: analytical characterization only.
Precision	Stability of HLA allele assignment should be demonstrated under repeatability and local intermediate precision conditions.	ISO 15189: validation design, competence, equipment and reagent control. ISO 5649: proportional validation when local modifications affect precision conditions.	C1: verify; C2: validate modified conditions; C3-4: full validation; C5: fit-for-purpose only.
Coverage, read depth and quality metrics	Adequate read depth, quality reads, locus review and acceptance/rejection criteria must be empirically established.	ISO 15189: quality control, validity of results and acceptance criteria. ISO 5649: performance specifications for adapted panels, targets or sequencing conditions.	All clinical categories: core; intensity increases from local verification to full/end-to-end validation.
Allele balance	Allele representation must be controlled to identify drop-out risk, imbalance, mixed signals or locus-specific technical variability.	ISO 15189: process control, result validity and risk-based review. ISO 5649: local validation when allele-balance claims are affected by adapted samples, targets or bioinformatics.	All clinical categories: core for informative heterozygous diploid loci; adapted claims require expanded validation.
Ambiguity management and phasing	P-group and non-P-group ambiguity, phasing support and reporting rules must follow HLA-specific requirements.	ISO 15189: result review, authorization and reporting limitations. ISO 5649: reporting limitations for laboratory-adapted or off-IFU claims.	All clinical categories: core; C5: no diagnostic reporting unless reclassified and validated.
Software validation and database control	Bioinformatics process, HLA database version, analysis rules, manual review and update/revalidation must be controlled.	ISO 15189: information management, software validation, change management and data integrity. ISO 5649: lifecycle control for laboratory-developed, laboratory-adapted or customized bioinformatics components.	All categories: required. C2-4 require impact assessment and proportional/full revalidation after changes.
Additional outputs and risk-based claims	Non-classical loci, CNV/HLA-loss signals, additional targets and non-standard samples require dedicated validation if reported.	ISO 15189: scope definition, risk management and reporting governance. ISO 5649: dedicated validation for laboratory-developed, laboratory-adapted, RUO-to-clinical or off-IFU claims.	C1: not reportable unless covered/verified; C2-4: dedicated validation; C5: exploratory only.
Reporting, EQA/PT and lifecycle monitoring	HLA reports, EQA/PT participation, ongoing monitoring and revalidation after changes are central to controlled H&I testing.	ISO 15189: reporting, validity of results, external quality assessment, monitoring and continual improvement. ISO 5649: lifecycle monitoring and change management for laboratory-developed or adapted workflows.	All clinical categories: core. C5: internal governance and explicit non-reportability.

### 3.3 Core verification and validation modules

#### 3.3.1 Concordance and agreement

Concordance should be assessed by comparison with reference or previously established HLA typing results. Concordance should be calculated at the locus and allele level according to predefined rules (Wittig et al., 2015; Bettinotti et al., 2018; Clinical and Laboratory Standards Institute. MM09-Ed3, 2023; Clinical and Laboratory Standards Institute, 2023). P-group ambiguities may be considered acceptable when they do not affect the intended reporting resolution or clinical interpretation (Clinical and Laboratory Standards Institute. MM09-Ed3, 2023; Clinical and Laboratory Standards Institute, 2023; European Federation for Immunogenetics, 2026; American Society for Histocompatibility and Immunogenetics. Standards fo r Accredited Laboratories, 2025).

Apparent discrepancies should be classified as true discordance, acceptable ambiguity, or technical deviation. This distinction is important because not all discrepancies have the same analytical or clinical meaning. Technical deviations associated with non-compliant input, failed QC or documented pre-analytical issues should be managed as validation nonconformities and samples repeated when appropriate.

For qualitative HLA genotype assignment, the primary metric should be reported as concordance or overall agreement rather than diagnostic accuracy, unless the study is specifically designed with a clinical reference standard and binary clinical endpoints (Clinical and Laboratory Standards Institute, 2023).

Overall agreement or concordance (%) = (number of concordant evaluable allele calls or loci/total number of evaluable allele calls or loci) × 100.

The calculation level, allele-level or locus-level, should be predefined. Non-evaluable loci, non-reportable loci and results excluded according to predefined nonconformity criteria should be reported separately.

#### 3.3.2 Precision

Precision should be evaluated through intra-run repeatability and inter-run reproducibility or intermediate precision. For local validation, inter-run studies usually represent intermediate precision rather than full inter-laboratory reproducibility. The primary endpoint should be allelic concordance; secondary endpoints can include variability in coverage, allele balance and run-level QC metrics (Wittig et al., 2015; Clinical and Laboratory Standards Institute. MM09-Ed3, 2023; Clinical and Laboratory Standards Institute, 2014).

Repeatability agreement (%) = (number of identical allele calls under repeatability conditions/total number of compared allele calls) × 100.

Intermediate precision agreement (%) = (number of identical allele calls across runs, operators, instruments or reagent lots/total number of compared allele calls) × 100.

CV% = (standard deviation/mean) × 100.

CV% should not be used as the primary precision endpoint for qualitative allele assignment, but only for quantitative quality indicators such as read depth or allele balance.

#### 3.3.3 Coverage

Coverage should be assessed separately for critical exons and broader targeted regions. The validation plan should define target read depth, minimum acceptable depth, proportion of loci or regions required to meet thresholds, recurrent low-performing regions, criteria for repeating analysis, and manual review rules. Deviations between target and minimum coverage may be acceptable only if allele assignment, resolution and final interpretation are not affected, and the review is documented (Wittig et al., 2015; Clinical and Laboratory Standards Institute. MM09-Ed3, 2023; European Federation for Immunogenetics, 2026; Jennings et al., 2017).

Critical-exon coverage compliance (%) = (number of critical exons meeting the predefined read-depth threshold/total number of evaluable critical exons) × 100.

All-target coverage compliance (%) = (number of targeted regions meeting the predefined read-depth threshold/total number of evaluable targeted regions) × 100.

Minimum coverage compliance (%) = (number of evaluable regions meeting the minimum read-depth threshold/total number of evaluable regions) × 100.

These metrics should be calculated separately for critical exons and all targeted regions when applicable.

#### 3.3.4 Allele balance

Allele balance should be evaluated in heterozygous diploid loci, excluding homozygous loci and non-informative regions. It is a key indicator in hybrid-capture workflows because imbalance may suggest preferential capture, reduced template quality, allele drop-out risk, mixed-template effects, or locus-specific technical variability. Laboratories should define actions for allele balance deviations, including review, test repeating, or restricted reporting.

Allele balance compliance (%) = (number of informative heterozygous diploid loci meeting the predefined AB criterion/total number of informative heterozygous diploid loci) × 100.

AB = lower allele read depth/higher allele read depth. Allele balance should be calculated only for informative heterozygous diploid loci or regions. Homozygous loci, non-informative regions, loci with known copy-number variability and samples with suspected mixed-template profiles should be excluded or interpreted using predefined review rules.

#### 3.3.5 Ambiguity management

Ambiguity rate should be evaluated separately for clinically acceptable ambiguities and potentially report-impacting ambiguities. The ambiguity policy should define how P-group and non-P-group ambiguities are managed, when additional testing is needed, and how residual ambiguity is reported. Software warnings, coverage gaps, and database version should be reviewed for ambiguous results (European Federation for Immunogenetics, 2026; American Society for Histocompatibility and Immunogenetics. Standards fo r Accredited Laboratories, 2025; Roy et al., 2018).

Ambiguity rate should be calculated only among concordant evaluated loci, after completion of the concordance assessment. Discordant, non-evaluable or technically excluded loci should be managed separately as discrepancies or validation nonconformities and should not be included in the ambiguity denominator. This differs from ambiguity rates calculated on the full dataset, as sometimes reported elsewhere, and should be stated clearly in the validation report.

P-group ambiguity rate (%) = (number of P-group ambiguous concordant loci/number of concordant evaluated loci) × 100.

Non-P-group ambiguity rate (%) = (number of non-P-group ambiguous concordant loci/number of concordant evaluated loci) × 100.

This approach allows ambiguity to be interpreted as residual resolution uncertainty within otherwise concordant HLA assignments.

#### 3.3.6 Run quality indicators

Run-level quality indicators may include Q30, percentage of reads identified, on-target percentage, barcode coefficient of variation, sample-level coverage distribution, allele balance distribution and software warnings. These indicators should be interpreted as a whole picture rather than in isolation. A single run metric outside a preferred threshold may trigger documented review rather than automatic data rejection, if sample and locus typing results remain compliant. The calculation rules for concordance, precision, coverage, allele balance, ambiguity, and mixed-template indicators are, therefore, reported directly within the corresponding validation sections, together with the relevant exclusion rules.

#### 3.3.7 Contamination and sample mix-up control

Sample mix-up and contamination are critical risks in NGS-based HLA typing and should be treated as core controls within a standards-based H&I framework. Low-level contaminating DNA may not always cause failure of standard QC metrics. Validation should therefore include control measures for sample tracking, barcoding or pooling, contamination prevention and review of unexpected alleles or warning patterns when clinically relevant (Clinical and Laboratory Standards Institute. MM09-Ed3, 2023; European Federation for Immunogenetics, 2026).

For controlled challenge experiments, the unexpected allele call rate (%) = (number of unexpected allele calls in a controlled challenge condition/total number of informative allele calls assessed) × 100.

The contaminant allele detection rate (%) = (number of detected contaminant-specific informative alleles/number of expected contaminant-specific informative alleles) × 100.

These metrics are specific to the experimental challenge model and should not be interpreted as diagnostic specificity, analytical sensitivity or contamination limit of detection.

### 3.4 Additional risk-based verification and validation modules

#### 3.4.1 Validated DNA input range and low-input robustness

Verification of acceptable performance within the manufacturer-defined DNA input range is required for routine implementation. Dedicated validation studies are needed when the laboratory intends to modify or extend that range, including lower-input conditions.

Serial DNA dilution input experiments should be interpreted as assessment of workflow robustness across DNA input concentrations and as definition of the minimum validated DNA input under predefined QC criteria. For qualitative constitutional HLA genotyping, this is not a classical analytical sensitivity or limit-of-detection study. The endpoint is a reliable genotype assignment, not the detection of a low-frequency analyte (Clinical and Laboratory Standards Institute. MM09-Ed3, 2023; Jennings et al., 2017).

Performance should be assessed using allelic concordance, average coverage, minimum coverage, allele balance, ambiguity rate, software flags, need for test repeating and impact on final allele assignment. The final statement should define the recommended input range, the minimum validated input under local conditions, and circumstances requiring cautious interpretation or test repeating.

#### 3.4.2 Alternative sample sources

Alternative matrices such as saliva, buccal swabs, spleen, or lymph node tissue should be validated or verified before clinical use, when included in the intended use of the laboratory. These sample types may introduce lower DNA yield, variable DNA quality, storage-related degradation, and increased pre-analytical variability. If the matrix is not included in the manufacturer’s IFU, it should be treated as an off-IFU or laboratory-adapted component (International Organization for Standardization, 2024).

#### 3.4.3 Post-transplant mixed-profile samples

Post-transplant mixed-profile samples were defined as samples collected after allogeneic transplantation in which recipient- and donor-derived DNA may coexist, generating mixed-template HLA profiles. These samples were not intended for quantitative chimerism assessment, but were used as challenge conditions to evaluate software warnings, allele balance deviations, unexpected allele calls, linkage inconsistencies and reporting restrictions.

If such samples are accepted, the laboratory should validate or verify their interpretation rules, define review triggers, integrate chimerism information when available, and specify when reporting is restricted or not permitted.

#### 3.4.4 Software-derived CNV or HLA-loss signals

Some analysis software may provide copy number-related outputs or allele-specific imbalance indicators. These may be useful as qualitative challenge signals but should not be reported diagnostically unless the workflow has been specifically validated for that intended use. When not validated for formal reporting, CNV or HLA-loss outputs should be described as exploratory, qualitative, or internal review indicators only.

### 3.5 Software validation, database and bioinformatics control

Software validation and bioinformatics control are essential components of HLA NGS method validation. In NGS-based HLA typing workflows, including capture-based approaches, the analysis software may directly contribute to read alignment, allele assignment, phasing, ambiguity resolution, quality flagging, reference database matching and generation of reportable results. Therefore, the role of the software should be defined for each workflow and verified within the local laboratory environment according to the intended clinical use (Hosomichi et al., 2015; Wittig et al., 2015; International Organization for Standardization, 2022; Clinical and Laboratory Standards Institute. MM09-Ed3, 2023; Osoegawa et al., 2021). This approach is consistent with HLA-specific NGS validation guidance and broader diagnostic NGS recommendations, which treat bioinformatics, data management and software-dependent interpretation as controlled components of the validated examination procedure (European Federation for Immunogenetics, 2026; American Society for Histocompatibility and Immunogenetics. Standards fo r Accredited Laboratories, 2025; Matthijs et al., 2016).

At minimum, the laboratory should document the software name and version, HLA reference database version, analysis parameters, reportable loci, allelic ambiguity management rules, manual review criteria, reporting rules and conditions requiring analysis repetition and trained staff interpretation. Validation or verification should demonstrate that the software supports accurate and traceable HLA assignment using reference or previously characterized samples, correctly manages homozygous and heterozygous loci, identifies relevant deviations from quality criteria and allows review of key indicators such as coverage, allele balance and unresolved ambiguities. HLA-specific guidance also emphasizes validation of data-management systems, run acceptance metrics and independently validated software used to generate genotyping information (Weimer et al., 2016; International Organization for Standardization, 2024). General NGS bioinformatics guidance similarly supports documented pipeline design, validation, operation and oversight by qualified personnel (Clinical and Laboratory Standards Institute. MM09-Ed3, 2023; Roy et al., 2018; Osoegawa et al., 2021).

Because HLA allele assignment depends on the reference database used, database version control should be included in validation, routine monitoring and change-management procedures. Verification and validation should be performed using reference or previously characterized DNA samples with established HLA typing results, allowing direct assessment of concordance. The identification of potentially novel alleles is instead managed within routine quality assurance through yearly IPD-IMGT/HLA Database updates and software-generated mismatch or sequence-discrepancy alerts, followed by expert review according to the laboratory’s established procedures. Where needed, previously characterized datasets or *in silico* data may supplement re-verification of software or bioinformatics pipeline changes, provided that their intended purpose and limitations are documented (Wittig et al., 2015; Clinical and Laboratory Standards Institute. MM09-Ed3, 2023; European Federation for Immunogenetics, 2026; Roy et al., 2018; Osoegawa et al., 2021; Duncavage et al., 2023).

Manual review remains essential, particularly in the presence of low-coverage regions, borderline allele balance, non-P-group ambiguities, mixed profiles, unexpected linkage patterns or software-generated warnings. The laboratory should define which outputs are reportable, which require confirmation and which should be treated as exploratory or internal quality signals. Software-derived outputs that have not been validated for clinical reporting, such as CNV-like signals when available, should not be included in the diagnostic report unless a dedicated validation has been performed for that specific intended use. This is consistent with diagnostic NGS guidance requiring transparent reporting rules, controlled interpretation and documented management of bioinformatics outputs (Roy et al., 2018; Matthijs et al., 2016).

Software validation documentation should include evidence of user training, version traceability, data review procedures, criteria for manual override, deviation management, reanalysis rules and final result approval. In this way, the bioinformatics component is controlled as an integral part of the validated examination procedure (International Organization for Standardization, 2022; International Organization for Standardization, 2024).

### 3.6 Risk management and reporting governance

The complete workflow, pre-examination, examination and post-examination phases, should be covered by risk analysis. It should be proportional to the intended use and clinical impact of the result. Risk control measures should be implemented, and residual risk should be reviewed before routine implementation. When a semi-quantitative matrix is used, the calculation model should be stated in the validation plan. A simple risk score may be calculated as (probability score × severity score); when detectability is included, the risk priority number may be calculated as (probability score × severity score × detectability score). The selected model should follow the laboratory risk-management procedure and residual risk after mitigation should be documented. Numerical scores should support prioritization but should not replace documented expert review of residual risk. Representative risk examples across the workflow steps are summarized in Table 3.

**TABLE 3:** Risk management examples across the NGS HLA typing workflow.

Workflow step	Potential risk	Possible control
Pre-examination	Sample misidentification, inadequate matrix, low quantity or poor DNA quality	Sample acceptance criteria, traceability, DNA quantification and quality review
Library preparation	Pipetting error, cross-contamination, index/UDI contamination	Unidirectional workflow, trained personnel, filter tips, plate maps and documented controls
Hybrid capture	Suboptimal enrichment, timing or temperature deviations	Strict timing control, calibrated equipment, validated protocol conditions
Sequencing	Run failure, barcode imbalance, low on-target rate	Run QC thresholds, trend monitoring and documented review
Software analysis	Misassignment, ignored warnings, database-related ambiguity	Version control, software verification, trained reviewer and manual review checklist
Post-examination	Over-reporting non-validated targets, incorrect interpretation, inappropriate manual override	Defined reporting scope, authorization rules, interpretative governance and nonconformity management
Lifecycle	Software, reagent, platform or database changes affecting performance	Change management, verification/revalidation criteria and periodic review

The reportable data scope should be explicitly defined. Laboratories should state which loci, resolution levels and sample types are clinically validated. Any non-classical HLA, loci, copy number variation signals, alternative matrices or exploratory outputs should be excluded from diagnostic reporting, unless specifically validated and included in the intended use.

Interpretative governance should define who may authorize results, who may perform manual review, how ambiguities are resolved, how discordant or suspicious results are managed, and when additional testing or analysis repeating is required. These rules should be documented in the validation report and in the routine operating procedure.

Roles and responsibilities for validation planning, analytical execution, bioinformatic review, discrepancy assessment, approval of validation evidence, clinical result authorization and change management should be predefined and documented. A responsibility matrix may be used to distinguish personnel responsible for performing, reviewing and approving each activity, according to documented competence and authorization.

### 3.7 Implementation, monitoring and change management

Validation should not end with the initial performance report. A routine monitoring plan should be implemented to confirm that the workflow continues to meet performance specifications. Monitoring indicators may include run failure rate, repeat rate, Q30, reads identified, on-target percentage, barcode CV, coverage, allele balance, ambiguity rate, nonconformities, EQA/proficiency testing performance and user feedback (International Organization for Standardization, 2022; Clinical and Laboratory Standards Institute. MM09-Ed3, 2023; European Federation for Immunogenetics, 2026; Jennings et al., 2017).

Sample failure rate (%) = (number of samples not meeting predefined reportability criteria/total number of tested samples) × 100.

Repeat testing rate (%) = (number of samples requiring repeat testing/total number of tested samples) × 100.

Run failure rate (%) = (number of runs not meeting predefined run acceptance criteria/total number of runs performed) × 100.

Monitoring indicators should be periodically trended by sample type, DNA input, run, operator, reagent lot, instrument, software version and database version.

Change management should define which changes require verification or revalidation. Minor changes may require limited verification, whereas significant changes such as new sample type, software pipeline, database strategy, sequencing platform, reportable locus set or clinical claim may require partial or full revalidation.

### 3.8 Practical checklist

The core and additional modules described above are consolidated into a practical checklist, summarizing the main question and expected documentation output for each module (Table 4).

**TABLE 4:** Practical checklist for validation and implementation documentation.

Module	Core or optional	Main question	Documentation output
Intended use	Core	What is the clinical purpose and reportable scope?	Intended-use statement and reporting policy
Workflow classification	Core	Is the workflow IVDR, RUO, off-IFU or laboratory-adapted?	Regulatory/standards applicability assessment
Concordance/agreement	Core	Does the method assign the expected HLA genotype?	Concordance table and discrepancy review
Precision	Core	Are results stable within and between runs?	Repeatability/intermediate precision results
Coverage	Core	Are critical regions sufficiently covered?	Coverage summary and review rules
Allele balance	Core	Is allele representation adequate?	Allele balance summary and actions for deviations
Ambiguity	Core	How are unresolved combinations managed?	Ambiguity policy and examples
Run QC	Core	Are run and sample QC criteria defined?	Run/sample/locus acceptance criteria
Contamination/sample mix-up control	Core	How are contamination, sample tracking and sample interchange risks controlled?	Contamination controls, traceability review and challenge-model indicators when applicable
Software validation/database control	Core	Are analysis versions and rules validated and controlled?	Software validation and database control record
Risk management	Core	What can fail and how is it controlled?	Risk analysis and residual risk review
Reporting governance	Core	Which outputs are clinically reportable?	Reporting scope, authorization rules and limitations
Roles and responsibilities	Core	Who performs, reviews and approves each validation and reporting activity?	Responsibility and authorization matrix
EQA/PT strategy	Core	How is external performance verified?	EQA/PT participation and review records
DNA input	Core for verification; additional validation if adapted	Does the workflow perform acceptably within the IFU-defined DNA input range, and has any locally modified or extended range been validated?	Local verification of IFU-defined input conditions; dedicated validation of any modified or extended range
Alternative matrices	Optional/adapted	Can non-standard sample types be used?	Matrix-specific validation/verification
Post-transplant mixed-profile samples	Optional/risk-based	How are donor/recipient mixed-template HLA profiles managed?	Review triggers, reporting restrictions and challenge evidence
CNV/HLA-loss signals	Optional/adapted	Are these clinically reportable or exploratory only?	Explicit limitation or dedicated validation
Monitoring/change control	Core	How is performance maintained over time?	CQI plan and change management procedure

## 4 Practical implementation example

To illustrate the applicability of the framework, it was applied to an internal validation dataset generated under routine clinical laboratory conditions using a commercial full-length HLA capture-based next-generation sequencing workflow. The dataset was used as a worked implementation example and not as a product-specific comparative performance claim.

The implementation dataset included reference and proficiency testing samples, blind samples, intra-run and inter-run replicates, serial DNA input dilution experiments, simulated sample mix-up experiments and post-transplant mixed-template challenge samples. The dataset also included qualitative review of software-derived copy number-related signals and an HLA-loss challenge sample (Bettinotti et al., 2018). Detailed data are reported in Supplementary Appendix A as a workflow implementation example.

## 5 Discussion

A modular framework for the verification and validation of hybrid-capture HLA NGS workflows in clinical histocompatibility laboratories is proposed in this work. The main value of the framework is to provide a transferable structure for defining the appropriate extent of verification or validation according to intended use, workflow category, local adaptations, clinical risk and reportable scope.

The main contribution of the framework is the translation of standards-based requirements into a structured, risk-based implementation pathway. Rather than applying a uniform validation scheme to all HLA NGS workflows, the proposed approach links the extent of verification or validation to the intended use, regulatory status, degree of local modification, reportable scope and associated clinical risk.

A central point of the framework is the distinction between clinically reportable outputs and technically available outputs not validated for diagnostic use. In hybrid-capture workflows, the software may generate information beyond the primary HLA typing claim. Such outputs may be useful for internal review or future development, but should not be included in diagnostic reports without dedicated validation.

Software and analytical continuous monitoring are also central to the framework. In HLA NGS workflows, software, the HLA reference database, analysis parameters, manual review rules, and version updates may directly influence allele assignment, phasing, ambiguity resolution, and reporting. These components should therefore be managed through documented validation or verification, version control, change-impact assessment, and post-implementation monitoring.

Published studies on the implementation and validation of HLA NGS methods have evaluated several of the performance characteristics included in the present framework: concordance, precision, sequencing coverage, ambiguity resolution, quality control, and bioinformatic performance (Weimer et al., 2016; Clinical and Laboratory Standards Institute. MM09-Ed3, 2023). More recent studies have highlighted the increasing detection of novel HLA alleles by NGS and the need for standardized approaches to their verification, reporting, and submission to the IPD-IMGT/HLA Database (Tran et al., 2023). The continued expansion and regular updating of the HLA reference database further support the importance of database-aware interpretation and controlled management of database changes (Robinson et al., 2024). In parallel, long-term external proficiency testing experience supports the role of continued external quality assessment in maintaining high-resolution HLA typing performance (Voorter et al., 2024). The present framework is consistent with these observations and brings these analytical, bioinformatic, reporting, and quality-management elements together within a modular verification and validation approach.

The applicability of the framework under routine conditions is illustrated by the implementation example; however, these data should be interpreted as local implementation evidence rather than as universal performance claims. The main limitations of the proposed framework are the need for adaptation to the local regulatory and accreditation context, the absence of universal numerical acceptance thresholds, and the requirement for dedicated validation when laboratories introduce extended claims, alternative matrices, post-transplant mixed-profile challenge samples, CNV/HLA-loss signals, non-classical loci, or customized bioinformatics.

## 6 Conclusion

Hybrid-capture HLA NGS validation should be guided by intended use, workflow category, local adaptation, clinical risk and reportable scope. A commercial IVDR-regulated workflow used according to the manufacturer’s IFU, an IVDR-regulated workflow with local adaptations, an RUO workflow implemented for clinical reporting and a laboratory-developed or laboratory-adapted workflow require different levels of verification or validation, but should be managed within a common quality and risk-based structure.

The proposed modular framework provides a transferable approach for defining core validation modules, activating additional risk-based modules when specific claims are introduced, and distinguishing clinically reportable outputs from exploratory or non-validated outputs. By integrating analytical performance, software validation, database control, reporting governance, risk management, post-validation monitoring and change management, the framework supports controlled implementation and lifecycle management of hybrid-capture HLA NGS workflows in clinical histocompatibility laboratories.

## Data Availability

The raw data supporting the conclusions of this article will be made available by the authors, without undue reservation.

## References

[B1] American Society for Histocompatibility and Immunogenetics. Standards for Accredited Laboratories (2025). Revised Standards. Mount Laurel, NJ. American Society for Histocompatibility and Immunogenetics.

[B2] Bettinotti M. P. Ferriola D. Duke J. L. Mosbruger T. L. Tairis N. Jennings L. (2018). Characterization of 108 genomic DNA reference materials for 11 human leukocyte antigen loci: a GeT-RM collaborative project. J. Mol. Diagn 20 (5), 703–715. 10.1016/j.jmoldx.2018.05.009 29959025 PMC6939753

[B3] Clinical and Laboratory Standards Institute (2014). EP05-A3. Evaluation of Precision of Quantitative Measurement Procedures; Approved Guideline. 3rd ed. Wayne, PA: Clinical and Laboratory Standards Institute.

[B4] Clinical and Laboratory Standards Institute (2023). EP12. Evaluation of Qualitative, Binary Output Examination Performance. 3rd ed. Wayne, PA: Clinical and Laboratory Standards Institute.

[B5] Clinical and Laboratory Standards Institute. MM09-Ed3 (2023). Human Genetic and Genomic Testing Using Traditional and High-Throughput Nucleic Acid Sequencing Methods. 3rd ed. Wayne, PA: Clinical and Laboratory Standards Institute.

[B6] Duncavage E. J. Coleman J. F. de Baca M. E. Kadri S. Leon A. Routbort M. (2023). Recommendations for the use of *in silico* approaches for next-generation sequencing bioinformatic pipeline validation: a joint report of the Association for Molecular Pathology, Association for Pathology Informatics, and College of American Pathologists. J. Mol. Diagn 25 (1), 3–16. 10.1016/j.jmoldx.2022.09.007 36244574

[B7] European Federation for Immunogenetics (2026). Standards for Histocompatibility & Immunogenetics Testing. Alice Scarpa, Piacenza: European Federation for Immunogenetics.

[B8] European Parliament and Council (2017). Regulation (EU) 2017/746 of 5 April 2017 on in vitro Diagnostic Medical Devices and Repealing Directive 98/79/EC and Commission Decision 2010/227/EU.Official J. Eur. Union

[B9] Hosomichi K. Shiina T. Tajima A. Inoue I. (2015). The impact of next-generation sequencing technologies on HLA research. J. Hum. Genet. 60 (11), 665–673. 10.1038/jhg.2015.102 26311539 PMC4660052

[B10] International Organization for Standardization (2022). Medical Laboratories—Requirements for Quality and Competence. Geneva: ISO. 10.1111/j.1423-0410.2002.tb05329.x 12617164

[B11] International Organization for Standardization (2024). ISO 5649:2024. Medical Laboratories—Concepts and Specifications for the Design, Development, Implementation and Use of laboratory-developed Tests. Geneva: ISO.

[B12] Jennings L. J. Arcila M. E. Corless C. Kamel-Reid S. Lubin I. M. Pfeifer J. (2017). Guidelines for validation of next-generation sequencing-based oncology panels: a joint consensus recommendation of the Association for Molecular Pathology and College of American Pathologists. J. Mol. Diagn 19 (3), 341–365. 10.1016/j.jmoldx.2017.01.011 28341590 PMC6941185

[B13] Matthijs G. Souche E. Alders M. Corveleyn A. Eck S. Feenstra I. (2016). Guidelines for diagnostic next-generation sequencing. Eur. J. Hum. Genet. 24 (1), 2–5. 10.1038/ejhg.2015.226 26508566 PMC4795226

[B14] Osoegawa K. Montero-Martin G. Mallempati K. C. Mack S. J. Milius R. P. Maiers M. (2021). Challenges for the standardized reporting of NGS HLA genotyping: surveying gaps between clinical and research laboratories. Hum. Immunol. 82 (11), 820–828. 10.1016/j.humimm.2021.08.011 34479742 PMC8545853

[B15] Robinson J. Barker D. J. Marsh S. G. E. (2024). 25 years of the IPD-IMGT/HLA database. HLA 103 (6), e15549. 10.1111/tan.15549 38936817

[B16] Roy S. Coldren C. Karunamurthy A. Kip N. S. Klee E. W. Lincoln S. E. (2018). Standards and guidelines for validating next-generation sequencing bioinformatics pipelines: a joint recommendation of the Association for Molecular Pathology and the College of American Pathologists. J. Mol. Diagn 20 (1), 4–27. 10.1016/j.jmoldx.2017.11.003 29154853

[B17] Tran J. N. Sherwood K. R. Mostafa A. Benedicto R. V. ElaAlim A. Greenshields A. (2023). Novel alleles in the era of next-generation sequencing-based HLA typing calls for standardization and policy. Front. Genet. 14, 1282834. 10.3389/fgene.2023.1282834 37900182 PMC10611506

[B18] Voorter C. E. M. Groeneveld L. Heidt S. Wieten L. (2024). Evaluation of 19 years of international external proficiency testing for high-resolution HLA typing. Front. Genet. 14, 1290915. 10.3389/fgene.2023.1290915 38348410 PMC10859402

[B19] Weimer E. T. Montgomery M. Petraroia R. Crawford J. Schmitz J. L. (2016). Performance characteristics and validation of next-generation sequencing for human leucocyte antigen typing. J. Mol. Diagn 18 (5), 668–675. 10.1016/j.jmoldx.2016.03.009 27376474

[B20] Wittig M. Anmarkrud J. A. Kässens J. C. Koch S. Forster M. Ellinghaus E. (2015). Development of a high-resolution NGS-based HLA-typing and analysis pipeline. Nucleic Acids Res. 43 (11), e70. 10.1093/nar/gkv184 25753671 PMC4477639

