# Supplementary material for: A modular verification and validation framework for hybrid-capture next-generation sequencing HLA typing in clinical histocompatibility laboratories
Source: Front Genet. 2026 Oct 12;17:1959391. doi: 10.3389/fgene.2026.1959391 (PMC13696030; doi:10.3389/fgene.2026.1959391)
Supplement: Supplementary file 1 [file Supplementaryfile1.docx]

***Appendix A. Workflow implementation example: One Lambda HybriType NGS assay***

This appendix provides an example of how the modular framework can be applied to a specific commercial hybrid-capture NGS HLA typing workflow. It is intended as an implementation example and not as a comparative performance claim for all capture-based products.

**A.1 Intended use and validation scope**

The assay was implemented for high-resolution HLA typing by NGS in HSCT patient and donor matching, with selected HLA-disease applications. Clinical reporting was limited to validated classical HLA loci. Non-classical HLA loci and CNV-related signals were treated as research or qualitative challenge outputs unless supported by a dedicated validation and reporting policy. This corresponds to workflow category C3 (RUO workflows implemented for clinical reporting) as defined in Table 1, with non-classical HLA loci and CNV-related outputs handled under C5 principles (exploratory use only, not clinically reportable) unless separately validated.

**A.2 Validation dataset**

The sample numbers reported in Table A.2 were not derived from a published sample-size calculation or intended to represent universal minimum requirements for HLA NGS validation. The size of the implementation dataset was primarily determined by the available analytical capacity of two 96-reaction kits, including negative controls. Within this practical constraint, sample selection and allocation to the different performance studies were based on the locally defined reportable scope, the routine case mix of the laboratory, and the analytical characteristics considered relevant to the intended clinical use of the workflow.

| **Component** | **Implementation example** |
| --- | --- |
| Overall design | 185 analyses across 8 sequencing runs, 2 operators and 2 Illumina MiSeq platforms |
| Accuracy | 112 EPT/reference samples and 10 blind EPT samples |
| Precision | Intra-run: 2 samples in triplicate; inter-run/intermediate precision: 3 samples across 4 runs and 1 sample across 2 runs |
| DNA input | 1 sample tested across 7 dilutions from 200 to 2.5 ng/uL; 5 samples tested across 3 dilutions from 200 to 1 ng/uL |
| Mixed-template assessment | Artificial sample mix-up models at 20%, 5% and 0.5% contaminating input; one post-haploidentical-HSCT mixed-profile EPT sample |
| Additional outputs | Qualitative CNV signal review on diploid loci and use of an artificial HLA-loss sample as a qualitative challenge sample |

**A.3 Quality metrics and acceptance criteria**

The numerical thresholds reported below are workflow-specific acceptance criteria used in this implementation, including manufacturer-defined QC metrics where applicable, and should not be interpreted as universal values. Coverage criteria are applied across the loci and targeted regions specified for each metric.

| **Metric** | **Example criterion** | **Use** |
| --- | --- | --- |
| Q30 | >=80% | Run-level sequencing quality indicator |
| Reads identified | >=90% | Run/sample assignment and target identification indicator |
| On-target reads | >=60% | Capture enrichment performance |
| Barcode CV | <=35% as preferred threshold | Values above threshold triggered documented review if sample/locus metrics remained acceptable |
| Exon 2 read depth | >=100x target; minimum >=50x | Evaluated as critical coverage component |
| All-exon read depth | >=100x in >=95% loci; minimum >=50x in >=95% loci | Evaluated across targeted regions |
| Allele balance | AB >=0.3 in >=95% informative heterozygous diploid loci | No allele drop-out accepted |

**A.4 Analytical performance summary**

Coverage analysis performed on samples with compliant DNA input (5–200 ng/µl). Allele balance analysis performed on 2,842 diploid heterozygous loci (AB >0), excluding homozygous loci and missing genomic regions values.

| **Endpoint** | **Result** | **Interpretation** |
| --- | --- | --- |
| Exon 2 coverage >=100x | 2997/3009 loci | 99.6% |
| Exon 2 minimum coverage >=50x | 3009/3009 loci | 100% |
| All-exon coverage >=100x | 2868/3009 loci | 95.3% |
| All-exon minimum coverage >=50x | 2973/3009 loci | 98.8% |
| Allele balance >=0.3 | 2781/2842 informative heterozygous diploid loci | 97.9% |
| Intra-run repeatability | 2 samples in triplicate | 100% allelic concordance |
| Inter-run/intermediate precision | 3 samples x 4 runs; 1 sample x 2 runs | 100% allelic concordance |
| Run Q30 | Range 85-92% | Compliant |
| Reads identified | Range 93-98% | Compliant |
| On-target reads | Range 62-75% | Compliant |
| Barcode CV | Range 17.08-53.72% | Variable; no associated impact on coverage or allele balance |

**A.5 Accuracy and ambiguity calculations**

Analytical accuracy was assessed by comparison with reference typing results (HLA database version 3.60.0.0). Concordance analysis was performed considering P-group ambiguities as acceptable. Ambiguity rate defined as the proportion of loci with unresolved allele combinations among accuracy-based sorted concordant loci. P-group ambiguities were considered separately from non-P-group ambiguities.

| **Dataset / endpoint** | **Calculation** | **Result / note** |
| --- | --- | --- |
| EPT/reference samples | 1207/1217 concordant loci | 99.18% concordance; discordance associated with documented low-input technical deviation and resolved on repeat |
| Non-P-group ambiguity | 3/1207 concordant evaluated loci | 0.25% |
| P-group ambiguity | 55/1207 concordant evaluated loci | 4.56% |

When nonconforming low-input samples were excluded or repeated according to predefined criteria, concordance of clinically reportable results was complete.

**A.6 Validated DNA input range and low-input robustness**

Serial DNA input dilution experiments were interpreted as validation of the locally accepted DNA input range and assessment of low-input robustness. Correct genotype assignments were maintained across the tested DNA input range, including low-input conditions below the recommended range. Minor deviations in coverage or QC metrics occurred at the lowest concentrations but did not affect final allele assignment in the tested samples.

**A.7 Mixed-template and contamination challenge**

Mixed-template and contamination experiments were treated as challenge conditions to characterize software behavior and warning patterns, not as formal diagnostic specificity or contamination limit of detection (LoD) studies. Artificial mixtures at 20%, 5% and 0.5% contamination, together with a post-haploidentical HSCT EPT sample with mixed chimerism, showed that standard QC metrics may remain acceptable despite mixed-template signals. Increased background, exon/intron mismatches, unexpected DRB3/4/5 linkage, allele balance deviations and CNV-related warnings were therefore considered review triggers requiring expert assessment, sample traceability review and, when appropriate, repeat or confirmatory testing. The false-positive allele call and contaminant allele detection rates reported below are specific to these challenge conditions and should not be generalized as assay-level diagnostic specificity claims.

| **Challenge condition** | **Informative alleles** | **False-positive allele rate** | **Contamination detection rate** | **QC values / System Comments** | **Interpretation** |
| --- | --- | --- | --- | --- | --- |
| Sample 1 + 20% Sample 2 | 19 | 10.53% (2/19) | 52.63% (10/19) | QC pass; high background; exon/intron mismatches; unexpected DRB345 linkage | Mixed profile |
| Sample 1 + 5% Sample 2 | 19 | 10.53% (2/19) | 10.53% (2/19) | Allele balance nonconformity; high background; exon/intron mismatches; unexpected DRB345 linkage | Low-level contamination |
| NTC + 0.5% Sample 2 | 34 | 0% (0/34) | 100% (34/34) | Minimum coverage nonconformity; high background; intronic mismatches | Contaminant-only profile |
| Post-haploidentical-HSCT mixed-profile sample | 7 | 28.57% | 42.86% | QC pass but warning pattern consistent with mixed template | Mixed profile |

**A.8 Exploratory CNV-related signal review**

CNV-related software outputs were evaluated as an exploratory qualitative module. Expected diploid loci were used to define locus-specific internal CNV distributions, excluding loci with known variable copy number such as DRB3/4/5 and HLA-H. Because CNV values were not centered on a universal value and showed locus-specific variability, interpretation was based on internal distributions rather than fixed thresholds. An artificial HLA loss sample was used as a qualitative challenge sample to assess the behavior of the algorithm. The sample showed allele balance nonconformity across multiple HLA loci and software-reported CNV discrepancies. These findings supported the use of CNV-related outputs as warning signals for expert review, but did not constitute validation for diagnostic HLA-loss reporting.

**A.9 Practical conclusion from the workflow implementation example**

The workflow implementation example demonstrated that hybrid-capture NGS HLA typing can be validated using a modular strategy that combines concordance, precision, coverage, allele balance, run quality control (QC), mixed-template assessment, DNA input robustness, software review, and risk management. It also illustrates why product-specific data should be reported as local implementation evidence rather than generalized performance claims for all NGS capture-based assays.
